# Effects of *Trichomonas gallinae* infection and diet on blood microbiome composition in european greenfinches (*Chloris chloris*)

**DOI:** 10.3389/fphys.2025.1576833

**Published:** 2025-06-05

**Authors:** Tatjana Krama, Ronalds Krams, Sergejs Popovs, Dita Gudrā, Maija Ustinova, Dāvids Fridmanis, Giedrius Trakimas, Jorge Contreras-Garduño, Dina Cīrule, Markus J. Rantala, Colton B. Adams, Priit Jõers, Indrikis A. Krams

**Affiliations:** ^1^ Department of Biodiversity, Institute of Life Sciences and Technology, Daugavpils University, Daugavpils, Latvia; ^2^ Chair of Plant Health, Institute of Agricultural and Environmental Sciences, Estonian University of Life Sciences, Tartu, Estonia; ^3^ Latvian Biomedical Research and Study Centre, Riga, Latvia; ^4^ Institute of Food Safety, Animal Health and Environment “BIOR ”, Riga, Latvia; ^5^ Institute of Biosciences, Life Sciences Center, Vilnius University, Vilnius, Lithuania; ^6^ Escuela Nacional de Estudios Superiores, National Autonomous University of Mexico, Morelia, Mexico; ^7^ Faculty of Veterinary Medicine, Latvia University of Life Sciences and Technologies, Jelgava, Latvia; ^8^ Department of Biology & Turku Brain and Mind Centre, University of Turku, Turku, Finland; ^9^ Department of Ecology and Evolutionary Biology, University of Tennessee, Knoxville, TN, United States; ^10^ Department of Psychology, University of Tennessee, Knoxville, TN, United States; ^11^ Institute of Molecular and Cell Biology, University of Tartu, Tartu, Estonia; ^12^ Department of Ecology, University of Latvia, Riga, Latvia; ^13^ Institute of Ecology and Earth Sciences, University of Tartu, Tartu, Estonia

**Keywords:** blood microbiome, *Chloris chloris*, European greenfinch, bird feeding behavior, health and disease ecology, trichomonosis outbreaks

## Abstract

Recent research has reported microbial invasion of the bloodstream in various disease-associated conditions. In this study, we investigated the role of trichomonosis outbreak (caused by the *Trichomonas gallinae* parasite) and food availability in shaping the blood microbiome composition of wintering greenfinches (*Chloris chloris*). Data were collected during two periods: before the outbreak (December) and during the outbreak (February). No bacterial contamination was observed in pre-epidemic blood samples. All individuals were infected during the outbreak, but greenfinches with irregular food access exhibited lower bacterial contamination in their blood. Individuals with permanent food access had a greater proportional representation of specific microbial taxa and higher alpha diversity in their blood microbiomes. However, beta diversity did not differ between the two groups. We demonstrated that trichomonosis infection and feeding regime play critical roles in mediating septic conditions of peripheral circulation during an outbreak, with food accessibility influencing blood microbial contamination. These findings integrate the impacts of feeding regimes and hematological responses to improve our understanding of the complex interactions between diet, disease, and physiological resilience in wild birds.

## Introduction

Populations of many animal species are declining as a result of global environmental changes. To understand the magnitude and causal mechanisms behind these population declines, it is crucial to evaluate health determinants such as diseases, parasites, immune responses, food availability and predictability, and stress resilience. Recent evidence suggests that the microbiome may serve as a meaningful indicator of wildlife health (Ribas et al., 2023). Multi-faceted approaches that include monitoring microbiome changes are necessary to track the health of wild animals, identify emerging threats, and implement effective conservation measures.

The microbiome refers to a dynamic community of microorganisms—including bacteria, archaea, fungi, algae, and protists—that inhabit specific environments with distinct physicochemical properties (Lederberg and McCray, 2001; Risely, 2020). These microbial communities are highly adapted to their respective habitats, and their composition and location are central to their function. Among microbiomes, the gut microbiome has garnered significant attention due to its critical role in health. Its diversity and function are shaped by diet, lifestyle, and numerous environmental factors (Thevaranjan et al., 2017). A diverse gut microbiome is associated with benefits such as improved digestion, optimized metabolism, enhanced immune responses, and protection against inflammation and disease. It also produces short-chain fatty acids, such as butyrate, which help maintain gut integrity and possess anti-inflammatory properties (Fusco et al., 2023).

However, compromised intestinal health—including increased gut permeability (i.e., “leaky gut”)—can allow microbes and harmful substances to enter the bloodstream. This process may trigger systemic health issues such as sepsis (Chancharoenthana et al., 2023) and reduced lifespan (Salazar et al., 2023). Recent studies suggest that impaired intestinal barriers in aged or stressed organisms contribute to systemic health deterioration and metabolic diseases (Rera et al., 2012; Clark et al., 2015; Egge et al., 2019).

Although blood has traditionally been considered sterile, microorganisms have been detected in the blood of healthy individuals (Potgieter et al., 2015; Castillo et al., 2019), leading to the concept of the blood microbiome (Qiu et al., 2019; Tan et al., 2023). While the existence and significance of the blood microbiome remain debated, the translocation of microbes from the gut to the bloodstream via compromised mucosal and epithelial barriers is a primary source of blood microbes (Camilleri, 2019). Microbes may also translocate from other surfaces, including the oral, nasal, and skin microbiomes (Cogen et al., 2008; Costello et al., 2019). Environmental stressors and diet exacerbate these mechanisms, shifting commensal microbes into pathogenic roles and contributing to altered blood microbial profiles (Païssé et al., 2016).

The role of stress, diet, and infection in shaping blood microbiomes has been explored in some non-human animals. For example, dietary-induced shifts in the gut and blood microbiome of dogs highlight the connection between gastrointestinal health and systemic circulation, even in healthy individuals (Scarsella et al., 2020). Microbial communities have been detected in the blood of other mammals and birds, though findings vary widely—from minimal microbial presence (Tan et al., 2023) to nearly universal detection (Damgaard et al., 2015; Païssé et al., 2016). Spatiotemporal factors such as seasonality and environmental conditions, as well as life history traits like behavioral and social adaptations, likely influence the extent of blood microbiome contamination. These interactions, spanning intracellular to ecological scales, could affect both the likelihood and magnitude of microbial translocation into the bloodstream.

Winter presents unique physiological challenges for resident birds. In cold climates, birds often accumulate substantial subcutaneous fat reserves to endure harsh conditions (Haftorn, 1989; Krams et al., 2010). However, a recent study on great tits (*Parus major*) revealed that birds with *ad libitum* access to sunflower seeds at permanent feeders exhibited higher body mass but suffered from reduced survival, slower reactions, and behaviors indicative of intestinal inflammation compared to birds with irregular food access (Krama et al., 2023). These findings suggest that excessive food availability may induce obesity and inflammation, leading to systemic health issues.

This study on the blood microbiome of European greenfinches (*Chloris chloris*) coincided with a trichomonosis epidemic affecting the local greenfinch population. Trichomonosis, caused by the protozoan parasite *Trichomonas gallinae*, is characterized by lesions in the oral cavity, esophagus, and crop of infected birds (Lawson et al., 2011). These lesions can impair food consumption, and lead to death. Moreover, lesions in the oral cavity and esophagus may facilitate microbial translocation into the bloodstream.

We hypothesized that infected greenfinches would exhibit a diverse microbial invasion into the blood, resulting from compromised epithelial barriers. Additionally, we predicted that permanent food access would be associated with a more diverse blood microbiome (both alpha and beta diversity) (Andermann et al., 2022) due to inflammation and health complications linked to excessive feeding and omega-6 fatty acid intake via consuming sunflower seeds (Simopoulos, 2016; Chang et al., 2021). By investigating the interactions between infection, diet, and blood microbiome composition in European greenfinches, this study aims to provide insights into the systemic effects of microbial translocation, offering a broader understanding of how environmental and physiological stressors influence wildlife health and conservation.

## Materials and methods

### Study areas and birds

This study was conducted near the town of Krāslava (55°53′N, 27°11′E) in southeastern Latvia during the winter of 2020/2021 (Rytkönen and Krams, 2003). Fieldwork took place in two independent areas of holiday cottages, located 5 km apart. Each area comprised approximately 80 summer cottages surrounded by coniferous forests interspersed with fields, bogs, and meadows near rivers and streams (Krama et al., 2023).

In each area, 4–5 feeders were installed, attracting 4–5 greenfinch flocks. Each flock consisted of 10–14 individual greenfinches that frequently used the same feeders. All birds were marked with aluminum and color bands, and no individuals were observed crossing the 5 km gap between the two feeding sites. Since the cottages were unoccupied during winter, no additional feeding occurred outside the study. This study focused on the body condition and blood microorganisms of adult male greenfinches, as they invest significantly in carotenoid-based plumage, which impacts health and immune function (Saks et al., 2003). Additionally, territorial males remained in the study area throughout winter. While data were collected from over 120 greenfinches during the study, the current analysis includes 35 individuals (18 males at permanent feeders and 17 males at irregular feeders) from which blood samples were obtained before and during the trichomonosis epidemic.

### Feeders and feeding regimes

Birds were divided into two groups: one group had access to permanent feeders offering unlimited sunflower seeds, while the other had access to feeders on an irregular schedule (2–3 times per week for <2 h per day). In one study area, feeders were continuously stocked with black oil sunflower seeds from mid-July until May of the following year under a constant feeding regime (Krama et al., 2023). In the second area, feeders were supplied twice weekly for a maximum of 2 h per session (irregular feeding).

### Microscopy detection of *Trichomonas gallinae*

The parasite *Trichomonas gallinae* is transmitted through direct contact and contaminated water, posing significant risks to avian populations. The epidemic persisted for 3 years and reduced the local greenfinch population by over 95%, as observed in other outbreaks affecting greenfinch populations (Lawson et al., 2011).

To assess the impact of *T. gallinae*, greenfinches were screened using oral cavity and crop swabs. Samples were examined immediately for motile trophozoites (Rijks et al., 2019). A total of 120 greenfinches were screened, including the 35 focal males (18 at permanent feeders and 17 at irregular feeders) before and during the epidemic.

Swabs and scrapings were collected from the oropharyngeal area and esophagus using sterile swabs, then processed as wet mounts in 0.5 mL of sterile saline (0.9%) for microscopic examination under a Zeiss light microscope. Trophozoites were identified by their rapid, jerky, non-directional motility and translucent, flagellated appearance, either solitary or in clusters. Slides were initially scanned under low magnification (100×), with detailed observations conducted at higher magnification (400×) to identify the parasite’s undulating membrane (Lehikoinen et al., 2013; Samour and Naldo, 2003).

*T. gallinae* was confirmed in all birds 1 month after the epidemic began, consistent with greenfinches’ social behavior and pathogen transmission at shared feeders.

### DNA extraction

DNA extraction from blood was performed using a modified phenol-chloroform method (Lugovska et al., 1999). In brief, the whole blood volume was diluted in 1 mL of ultra-pure water and transferred to a Lysing Matrix E tube (MP Biomedicals, United States). The samples were homogenized for 40 s (s) at a speed setting of 6.0, using a FastPrep-24 instrument (MP Biomedicals, United States), then centrifuged for 10 min at 14,100 rpm. The supernatant was transferred to a new tube, and 850 µL of cell suspension solution was added and mixed on a rotator for 5 min. Next, 100 µL of 10% SDS and 7.5 µL of Proteinase K (Thermo Fisher Scientific, United States) were added, mixed, and incubated at 50°C for 1 h. Following the incubation, 2 mL of phenol was added to the sample-reagent mixture and mixed on a rotator for 15 min. The samples were centrifuged for 10 min at 4,000 rpm, after which the supernatant was transferred to a new tube. Then, 2 mL of chloroform was added to the supernatant, mixed on a rotator for 5 min, and centrifuged for 10 min at 4,000 rpm. The supernatant was transferred to a new tube. To the supernatant containing the DNA fraction, 2 mL of isopropanol was added and gently mixed until the DNA precipitate formed. The samples were stored overnight at −20°C to facilitate DNA precipitation. The following day, the samples were centrifuged for 10 min at 4,000 rpm, and all liquid was discarded except for the precipitated DNA. Next, 5 mL of 70% ethanol was added, mixed for 1 min, and incubated for 2 min. The samples were centrifuged for 10 min at 4,000 rpm, and the ethanol was removed. The samples were dried at room temperature for 10 min or until all residual ethanol had evaporated. Finally, 100 µL of Low-TE buffer was added to the dry DNA samples, and the samples were placed on the rotator overnight. After this, the DNA was ready for downstream analysis.

### DNA extraction difficulties and consistency between sample sets

Both pre- and post-outbreak samples were processed using the same phenol–chloroform DNA extraction protocol, though with differences reflecting procedural optimization over time. The post-outbreak samples were extracted first and presented challenges due to the high content of host (avian) DNA and the nature of blood as a low-biomass sample. Initial attempts with commercial kits (e.g., FastDNA Spin Kit for Soil and NucleoMag Blood 200 µL) failed due to filter clogging and low yield, prompting a return to a modified phenol–chloroform protocol. While this method ultimately yielded usable DNA, PCR amplification was difficult and had to be repeated multiple times, leading to the loss of some samples. By the time the pre-outbreak samples were processed (approximately 1 year later), we had improved the phenol–chloroform protocol based on these experiences, enabling more efficient DNA extraction with fewer amplification problems. PCR conditions, indexing protocols, and downstream sequencing on the Illumina MiSeq platform were kept consistent across all samples to ensure comparability.

### *16S rRNA* V3−V4 gene amplification: blood samples collected before the onset of the trichomonosis outbreak

Amplifications and purifications of PCR products from the blood samples collected before the onset of trichomonosis epidemics were conducted using a Veriti® 96-Well Thermal Cycler (Applied Biosystems®, Thermo Scientific, Waltham, MA, United States). Samples were collected and stored at −80°C until processing. PCR amplification conditions included an initial denaturation at 98°C for 30 s, followed by 35 cycles of 98°C for 10 s, 67°C for 15 s, and 72°C for 15 s, with a final extension at 72°C for 7 min. Positive and negative controls were included to validate the results.

Following the manufacturer’s protocol, PCR products were purified to remove unincorporated primers and dNTPs using a QIAquick PCR Purification Kit (QIAGEN, Germany). The purified products were quantified using a NanoDrop™ 2000 spectrophotometer (Thermo Scientific, United States) and checked for quality on a 1.5% agarose gel stained with ethidium bromide. The gel was run at 100 V for approximately 30 min and visualized using a UV transilluminator. Despite following these rigorous protocols, no amplification of microorganism species was detected in any of the samples. This negative result suggests that the target organisms were absent in the collected samples or the conditions were not optimal for their detection.

### *16S rRNA* V3−V4 gene amplification and Illumina MiSeq sequencing: blood samples collected during the trichomonosis outbreak

Primers were designed for PCR amplification of the 16S rRNA V3−V4 region specific to the domain bacteria according to the 16S Metagenomic Sequencing Library Preparation protocol for Illumina MiSeq (Illumina Inc., United States). In brief, 10 ng of DNA was amplified by V3 (341F) and V4 (805R) primers using Phusion U Multiplex PCR Master Mix (Thermo Fisher Scientific, United States) with the following reaction conditions: denaturation at 98 °C for 30 s; 35 cycles of 98 °C for 10 s, 67 °C for 15 s, and 72 °C for 15 s; and fragment elongation at 72 °C for 7 min. Yield of PCR products was assessed using 1.2% agarose gel electrophoresis and purified using NucleoMag NGS Clean-Up and Size Select kit (Macherey-Nagel GmbH & Co. KG, Germany). Concentration of the PCR product was measured using Qubit dsDNA HS Assay Kit on Qubit 2.0 Fluorometer (Thermo Fisher Scientific, United States). During the second stage of PCR, 10 ng of V3 and V4 PCR product was used to add Illumina MiSeq i7 and i5 indexes using custom-ordered Nextera XT Index Kit (Illumina Inc., United States) primers (Metabion International AG, Germany). For this reaction, Phusion U Multiplex PCR Master Mix was used with thermal cycler reaction conditions as specified above. The 16S rRNA PCR products were then pooled and purified for the sequencing reaction using NucleoMag magnetic beads. The quality and yield of 16S rRNA V3−V4 amplicons were assessed using Agilent High Sensitivity DNA kit on Agilent 2,100 BioAnalyzer (Agilent Technologies, United States) and using Qubit dsDNA HS Assay Kit on Qubit 2.0 Fluorometer (Thermo Fisher Scientific, United States).

Before sequencing, all samples were diluted to 10 pM and pooled. Samples were paired-end sequenced using 500 cycles, using MiSeq Reagent Kit v2 on Illumina MiSeq. Each run was expected to produce at least 20,000 reads per sample. After the sequencing run was completed, individual sequence reads were filtered using MiSeq software to remove low-quality sequences.

### 16S rRNA sequence analyses

Sequence reads were quality filtered and trimmed using “fastp” v.0.23.4 with the quality threshold of Q20. All quality-approved sequences were imported into the QIIME2 v.2024.2 environment (Bolyen et al., 2019) for further analysis. The DADA2 plugin (Callahan et al., 2016) was used to pair forward and reverse reads and for extra sequence quality control and chimeric sequence removal using a pooled consensus method. The resulting feature table and sequences were used for *de novo* clustering, employing the vsearch plugin (Rognes et al., 2016) using a 97% identity threshold. Later, *de novo* multiple sequence alignment was performed using the MAFFT method (Katoh and Standley, 2013), while phylogenetic trees were constructed using FastTree2 (Price et al., 2010). *De novo* clustered sequences were used for taxonomic assignment with a pre-fitted sklearn-based taxonomy classifier based on the Silva v.138.1 99% identity reference database (Glöckner et al., 2017; SILVA, 2025) trained with naïve Bayes classifier (Pedregosa et al., 2011; Callahan et al., 2016).

### Contamination control procedures

To minimize the risk of contamination associated with low-biomass samples such as avian blood, we implemented strict contamination control procedures. Each DNA extraction batch included a negative extraction control (extraction blank) using nuclease-free water in place of blood. These blanks were subjected to all steps of the extraction protocol and processed alongside biological samples. Additionally, every PCR run included a no-template control (PCR negative control) to detect potential contamination during amplification. Sequencing data from all negative controls were carefully examined. Taxa consistently present in controls but absent or rare in experimental samples were flagged as potential contaminants and excluded from downstream analyses. These measures ensured that the detected microbial signatures in blood samples were unlikely to originate from laboratory reagents or environmental contamination.

### Statistical analyses

Before statistical analysis, rarefaction was employed to reach the resulting depth per sample of 6,300 sequences. Alpha diversity (observed, Chao1, Shannon, and Simpson indices) and beta diversity (Bray-Curtis distances) measures were calculated using the phyloseq v.1.46.0. Package (McMurdie and Holmes, 2013) in R v.4.3.2. Pairwise Wilcoxon rank-sum test with Holm’s *P*-value adjustment method was used to assess the significance of alpha diversity measurements between feeder types. Permutational multivariate analysis of variance (PERMANOVA) was performed to compare Bray-Curtis distances between feeder types.

**FIGURE 1:**
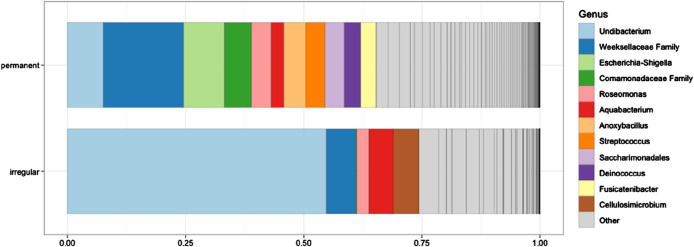
Relative abundance (%) of microbial taxa in the blood samples of greenfinches from irregular and constant feeding sites during winter. The 12 most abundant genera are shown. The different shades of gray within the “Other” category represent additional taxa that were not listed individually due to space constraints. None of the taxa differed significantly between the two bird groups (all *P* > 0.05).

Differential abundance testing was performed between feeder types to uncover microbial entities that might be attributed to the feeding type by MicrobiotaProcess v.1.14.0. Briefly, all taxonomic entities were evaluated to assess if values are distributed differently across taxonomic classes using the Kruskal–Wallis test at a significance threshold of 0.05. Then, significantly different taxonomic features were tested to determine if all pairwise comparisons between taxonomic subclasses within different classes follow a class trend using the Wilcoxon test at a significance threshold of 0.01. Significantly different taxonomic entities were evaluated using linear discriminant analysis (LDA). However, none of the microbial entities differed between the feeder types (Figure 1) and thus were not analyzed further.

## Results

### General profile of sequencing data

Illumina MiSeq sequencing of bacterial 16S rRNA amplicons from birds feeding at two types of feeders during the epidemic yielded a total of 1,526,627 raw reads. Pre-outbreak samples did not yield sequenceable amplicons, likely due to low bacterial DNA concentration and dominance of host DNA. After quality filtering and read merging, 977,272 high-quality sequences were retained. Among the samples, 17 permanent and 9 irregular feeders passed quality filtering, while 8 irregular feeders failed and were excluded from further analysis. These samples were excluded due to technical issues during DNA extraction, not low microbial content or rarefaction. After rarefaction and applying a 97% sequence similarity threshold, 2,128 bacterial OTUs were identified across all samples. Of these, 1,678 OTUs were found in samples from permanent feeders, and 450 OTUs were associated with irregular feeders, with a subset shared between both groups. The overall species richness coverage across all samples was 85.58% ± 11.33%, demonstrating that the sequencing depth was sufficient to capture the majority of bacterial taxa across the feeder types. Sample coverage was estimated using Good’s coverage index calculated via the ‘vegan’ R package. This index estimates the proportion of total taxa represented in each sample.

### Comparisons of microbial communities at two wintering sites during the trichomonosis epidemic: alpha and beta diversity

From a taxonomic perspective, the most abundant bacterial genus in irregular feeders was *Undibacterium* (54.8%), followed by unclassified representatives of the family *Weeksellaceae* (6.3%) and *Cellulosimicrobium* (5.5%). In permanent feeders, the most abundant taxa were unclassified representatives of *Weeksellaceae* (17.0%), the genus *Escherichia-Shigella* (8.7%), and *Undibacterium* (7.6%) (Figure 1).

**FIGURE 2:**
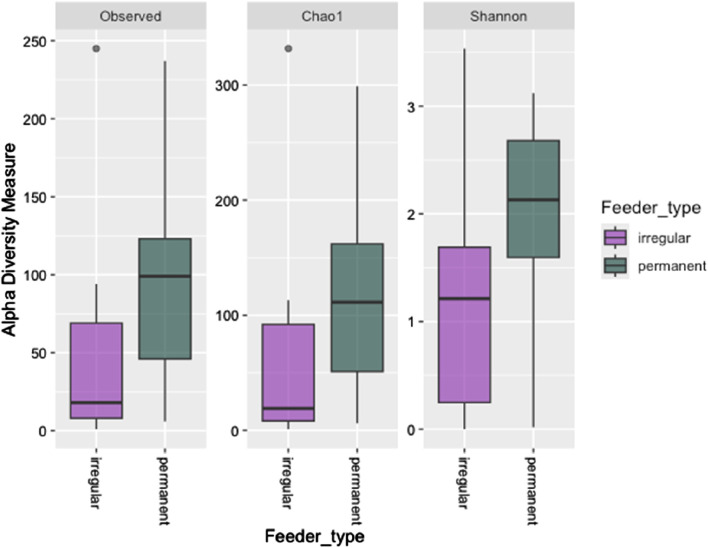
Alpha diversity indices (observed OTUs, Chao1, and Shannon diversity indices) of greenfinch blood samples in constant and irregular feeding locations.

**FIGURE 3:**
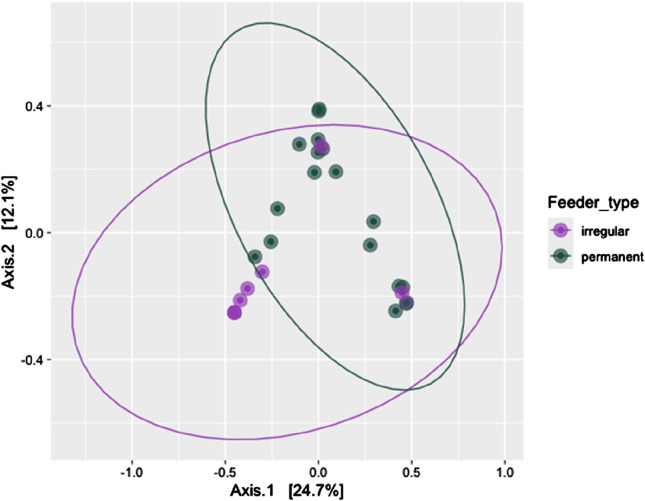
Beta diversity of the blood bacterial community of greenfinches at permanent and irregular feeders measured by permutational multivariate analysis of variance and compared as Bray-Curtis distances between feeder types.

Alpha diversity metrics, including observed richness (measured in OTUs), Chao1 (measured in OTUs), and the Shannon diversity index, were used to assess microbial richness and diversity across wintering sites. Significant differences in observed richness and Chao1 were found between the feeding sites (*P* = 0.027 and *P* = 0.034, respectively, Figure 2). However, the Shannon diversity index showed no significant difference in alpha diversity between the irregular and permanent feeding sites (*P* = 0.075, Figure 2). Comparisons of microbial community structure across the wintering sites did not reveal any clear separation in blood microbial community composition based on feeding regimes (Figure 2). Beta diversity, assessed using the Bray–Curtis dissimilarity matrix, supported this observation (Figure 3). PERMANOVA analysis showed no significant differences in bacterial community MDS distances between samples collected from irregular feeders and permanent feeders. Specifically, beta diversity distances based on the Bray–Curtis dissimilarity matrix were not significantly different between the two feeder types (*n* = 26, pseudo-F = 3.54, *P* = 0.072).

### Characterization of bacteria in blood samples

In blood samples collected during the epidemic, sepsis-causing bacteria such as *Streptococcus*, *Neisseria*, and *Escherichia-Shigella* were almost exclusively associated with permanent feeding sites. However, bacteria commonly linked to sepsis and capsule formation, such as *Haemophilus* and *Klebsiella*, were not detected. Most bacterial species identified were commensal or environmental in origin.

## Discussion

In this study, we investigated the effects of trichomonosis infection and food availability on blood microorganisms in uninfected and infected wintering greenfinches. Our findings reveal significant connections between parasite outbreaks, feeding conditions, and microbial translocation, shedding light on the intricate interplay between disease and diet.

Despite the absence of blood microbes in healthy (uninfected) birds at the study’s onset, microbial contamination of the bloodstream was detected in all birds after the epidemic began. This supports the hypothesis that severe disease and compromised health facilitate the translocation of gut or other commensal microbes into the bloodstream (Poore et al., 2020; Emery et al., 2021), challenging the concept of a normal blood microbiome (Tan et al., 2023). Notably, birds with permanent access to feeders exhibited greater microbial diversity in their blood than those with limited feeding access. Additionally, bacteria with high pathogenic potential, including capsule-forming antibiotic-resistant strains (Glenn et al., 2024), were predominantly found in birds with permanent food access. These findings align with prior research suggesting that excess low-quality food and sedentary lifestyles can exacerbate systemic health issues (Park et al., 2020; Cheng et al., 2023; Calella et al., 2024). Thus, sedentary behavior (as seen in greenfinches spending entire days near permanent feeders) and excessive energy reserves may impair physiological defenses, fostering microbial proliferation and, potentially, sepsis, in the context of immunocompromised hosts.

Our results indicate that excessive food availability impairs health and possibly increases vulnerability to infection-induced mortality. Birds exposed to high food availability likely suffered more severe oral cavity lesions, which may have facilitated systemic microbial translocation. This can be attributed to compromised mucosal and epithelial barrier integrity during disease or physiological/psychological stress, enabling the translocation of microorganisms into the bloodstream (Camilleri, 2019; Tan et al., 2023). Moreover, leaky gut syndrome cannot be ruled out in birds experiencing severe stress (Bischoff et al., 2014). These observations underscore the maladaptive consequences of excessive feeding under conditions of disease stress, suggesting a possible upper limit to the adaptive value of energy reserves.

The absence of bacterial DNA in blood samples collected before the infection confirms that the microorganisms detected during the epidemic were not sampling artifacts. The presence of a diverse microbial community in the blood of infected greenfinches, coupled with its absence in healthy individuals, supports the hypothesis of sporadic translocation of commensals—or their DNA—into the bloodstream. Many of the detected bacteria were commensal or environmental species, likely originating from food particles or drinking water. This suggests that much of the detected microbial DNA may be transiently translocated from organs such as the oral cavity, esophagus, or intestines rather than being endogenous to the blood.

This study also highlights the potential of blood microbiomes as markers of physiological stress and disease. The absence of microbes in the blood of healthy birds, combined with microbial contamination during severe infections, challenges the concept of a normal blood microbiome (Tan et al., 2023) and underscores the utility of blood microbial analysis as a sensitive indicator of health status in wild birds (Mandal et al., 2016). The presence of capsule-forming bacteria in the blood of heavily infected birds raises concerns about antibiotic resistance, which is also observed in wild animals (Laborda et al., 2022; Mourkas et al., 2024). Furthermore, the systemic impacts of microbial shifts associated with infection warrant further investigation. Future research should explore the mechanisms linking food availability and microbial translocation, particularly focusing on the roles of inflammation, immune function, and pathogen-induced anatomical damage.

In a broader context, these findings contribute to the growing evidence that modern environmental changes disrupt the ability of wild animals—and humans—to regulate body mass and energy reserves effectively (Speakman and Elmquist, 2022). Although fat storage is generally adaptive, our results suggest that modern conditions, characterized by food abundance and sedentary lifestyles, can lead to maladaptive outcomes, including obesity, weakened physiological responses to infections, inflammation, and sepsis.

The detection of diverse microbial taxa in the blood of greenfinches during the epidemic period, particularly in those at permanently stocked feeders, suggests that immune homeostasis may have been disrupted by pathogen pressure and environmental exposure. Stable and continuous access to high-quality food is known to buffer physiological stress responses by sustaining energetic reserves and modulating glucocorticoid levels. This stress-buffering effect may reduce gut permeability and lower the likelihood of microbial translocation from the gut or other mucosal surfaces into the bloodstream. Therefore, birds with regular access to feeders may maintain a more controlled inflammatory profile, allowing microbial signatures to reflect more stable interactions with host immunity rather than being driven purely by breakdown in barrier integrity. These patterns highlight the complex interplay between nutrition, infection, and immune regulation in shaping blood microbiome composition (Balmer et al., 2009; Criscuolo et al., 2008).

As blood is a low-biomass sample, the possibility of contamination is a valid concern (Brecher and Hay, 2005). However, our study incorporated extraction blanks and PCR negative controls, and the microbial taxa consistently identified in experimental samples were largely absent from these controls. Furthermore, the observed microbial profiles followed ecologically and physiologically meaningful patterns that aligned with feeding regime and infection status. For example, increased alpha diversity and dominance of certain genera in birds with permanent feeder access are consistent with expected patterns of microbial translocation or immune compromise. While no method can entirely eliminate the possibility of trace contamination, we believe the structured nature of our findings, together with rigorous control measures, supports the biological relevance of the detected blood microbiomes.

To further our understanding, future studies should (i) investigate the relationship between food availability, infection resistance, and the severity of microbial translocation in the bloodstream; (ii) examine the long-term evolutionary implications of food abundance and environmental changes on fat storage mechanisms and health outcomes in birds; (iii) explore interventions to mitigate the negative impacts of excessive food amounts, such as promoting natural foraging behaviors or regulating artificial feeding practices (Krama et al., 2023).

In summary, this study demonstrates that excessive food availability contributes to increased microbial diversity in the bloodstream, systemic health deterioration, and a higher risk of trichomonosis. Conversely, limited food availability may mitigate these risks. These findings underscore the importance of considering ecological and evolutionary contexts when assessing the adaptive value of food availability and the impacts of modern environmental changes. By linking infection, and environmental stressors, this research provides a foundation for future studies on the complex dynamics of health and survival in wild bird populations.

## Data Availability

The datasets presented in this study can be found in online repositories. The names of the repository/repositories and accession number(s) can be found below: https://www.ebi.ac.uk/ena, PRJEB85280.
